# Identification of novel urine proteomic biomarkers for high stamina in high-altitude adaptation

**DOI:** 10.3389/fphys.2023.1153166

**Published:** 2023-05-03

**Authors:** Chunlei Liu, Ge Guo, Xin Li, Yanying Shen, Xiang Xu, Yibing Chen, Hanlu Li, Jianxiu Hao, Kunlun He

**Affiliations:** ^1^ Translational Medicine Research Center, Medical Innovation Research Division of Chinese PLA General Hospital, Beijing, China; ^2^ Clinical Sample Bank, Medical Innovation Research Division of Chinese PLA General Hospital, Beijing, China; ^3^ Medical Big Data Research Center, Chinese PLA General Hospital, Beijing, China

**Keywords:** urine biomarkers, proteomic, high stamina, high-altitude adaptation, blood routine tests

## Abstract

**Introduction:** We aimed to identify urine biomarkers for screening individuals with adaptability to high-altitude hypoxia with high stamina levels. Although most non-high-altitude natives experience rapid decline in physical ability when ascending to high altitudes, some individuals with high-altitude adaptability continue to maintain high endurance levels.

**Methods:** We divided the study population into two groups: the LC group (low change in endurance from low to high altitude) and HC group (high change in endurance from low to high altitude). We performed blood biochemistry testing for individuals at high altitudes and sea level. We used urine peptidome profiling to compare the HH (high-altitude with high stamina) and HL (high-altitude with low stamina) groups and the LC and HC groups to identify urine biomarkers.

**Results:** Routine blood tests revealed that the concentration of white blood cells, lymphocytes and platelets were significantly higher in the HH group than in the HL group. Urine peptidome profiling showed that the proteins ITIH1, PDCD1LG2, NME1-NME2, and CSPG4 were significantly differentially expressed between the HH and HL groups, which was tested using ELISA. Urine proteomic analysis showed that LRG1, NID1, VASN, GPX3, ACP2, and PRSS8 were urine proteomic biomarkers of high stamina during high-altitude adaptation.

**Conclusion:** This study provides a novel approach for identifying potential biomarkers for screening individuals who can adapt to high altitudes with high stamina.

## Introduction

The high-altitude regions of plateaus are characterized by environmental factors such as hypoxia, cold temperatures, low pressure, and high radiation (Jeong et al., 2016). Each year, millions of people travel to high altitudes for various reasons including altitude training for athletes (Khodaee et al., 2016). Altitude training is often used to improve physical fitness and competitive performance in endurance sports such as middle-distance running, cross-country skiing, and cycling (Bailey et al., 1997). However, the results of altitude training are often uncertain, and not all athletes achieve the desired outcomes (Hamlin et al., 2018). Therefore, the question of why some individuals tolerate high altitudes and adapt well to high-altitude hypoxia training, whereas others do not remains unanswered. Additionally, individual athletes differ from each other. Every year during military screening, several individuals show high stamina at sea level, whereas at high altitudes, only some maintain high stamina, and others show rapid decline in stamina. This may be related to the characteristics specific to individuals. Therefore, the ability to screen for individuals who adapt well to high altitudes would be of great significance, particularly for military purposes.

To date, research on high-altitude adaptability has mainly focused on the genetic or biochemical differences between high-altitude residents such as Tibetans and sea-level residents, the physiological or other differences between individuals at sea level and high altitude, or the effect of altitude training on performance improvement (Simonson et al., 2010; Eichstaedt et al., 2020; Chiou et al., 2022). However, few studies have explored the mechanism of maintaining high performance during high-altitude adaptation. Recent advances in proteomics have enabled the identification of protein expression profiles in high stamina and low stamina individuals at high altitudes, providing a better understanding of the mechanisms underlying the functional adaptations of individuals in high-altitude hypoxic environments (Gracey et al., 2007).

This study aimed to screen for novel urine proteomic biomarkers for high-stamina in high-altitude adaptation. The study population was divided into two groups: the LC group (low change in endurance from low altitude to high altitude) and HC group (high change in endurance from low altitude to high altitude). We aimed to identify the differentially expressed proteins (DEPs) as potential biomarkers by comparing the generated urine peptidome profiles. In addition to military purposes, this research could be highly useful for screening individuals with good high-altitude adaptability in the sports field. Thus, identifying individuals with high physical fitness would be useful for choosing competitive personnel that can fulfil combat needs in plateau environments in a targeted manner.

## Materials and methods

### Study participants and sample collection

This study included 200 healthy Chinese men aged 18–25 years. The exclusion criteria included any health problems; known liver, lung, or cardiovascular disease; history of migraine or head injury; and smoking. None from the study population had prior experience at high altitudes. The study began with the participants undergoing 3,000 m training at sea level. Urine and blood samples were collected from these participants at sea level. They were then transported to high altitudes in Ali, Tibet (average elevation, >3,000 m). After 7 days of acclimatisation to high altitude (4,000 m), the participants underwent another 3,000 m training., and urine and blood samples were collected again. The participants were divided into HH (high-altitude with high stamina, n = 50), HL (high-altitude with low stamina, n = 50), LH (low-altitude with high stamina, n = 50), and LL (low-altitude with low stamina, n = 50) groups. Notably, collecting results for 3,000 m training for 200 participants at high altitudes (after only 7 days of acclimatisation) is quite difficult. Ethical approval for the study was obtained from the Chinese PLA General Hospital ethical committee (approval identifier S2019-035-01), and all protocols followed the established national and institutional ethical guidelines. All participants provided signed written informed consent.

### Routine blood tests

All blood samples collected from the HH and HL group participants at high altitude and LH and LL group participants at sea level were subjected to routine testing for haemoglobin (HGB), red blood cell (RBC) count, white blood cell (WBC) count, lymphocyte (Lym) count, neutrophil (Neu) count, monocyte (Mon) count, eosinophil (Eos) count, basophil (Bas) count, lymphocyte percentage (Lym%), monocyte percentage (Mon%), eosinophil percentage (Eos%), basophil percentage (Bas%), haematocrit (HCT), mean corpuscular volume (MCV), red blood cell distribution width coefficient of variation (RDW-CV), red blood cell distribution width standard deviation (RDW-SD), platelet count (PLT), mean platelet volume (MPV), platelet distribution width (PDW), and platelet volume (PCT). All routine blood test results were statistically analysed using SPSS software, and only the representative changes are shown in this paper.

### Urine proteome profiling and analysis

The urine samples were centrifuged at 12,000 × g for 30 min at 4°C, and the pellets were removed. The protein concentration in each sample was measured using the Bradford protein assay. The proteins were then digested with trypsin (Promega, United States), and 100 μg of the protein sample was loaded onto a 10 kDa filter unit (Pall, United States). The protein solution was reduced with 4.5 mM DTT for 4 min at 95°C. The proteins were digested with trypsin (enzyme-to-protein ratio of 1:50) overnight at 37°C. Formic acid (10%) was added to obtain a final formic acid concentration of 1%. The supernatant was centrifuged and the pH was adjusted to 10 with ammonia water. The digested samples were desalted, and different gradient eluents were configured to manually divide the samples into six components. They were rotary evaporated to dryness at 60°C for 90 min, reconstituted with formic acid, and placed in the machine for analysis.

### LC–MS/MS setup for DDA

An Orbitrap Fusion Lumos Tribrid mass spectrometer (Thermo Scientific, Germany) coupled with an Ultimata 3000-HPLC system (Thermo Scientific, Germany) was used for analysis. Each peptide sample was dissolved in 0.1% formic acid, and 1 µg of peptide was loaded onto a reversed-phase trap column (75 μm × 2 cm, 3 μm, C18, 100 Å, Thermo Scientific). The eluent was then transferred to a reversed-phase analytical column (50 μm × 500 mm, 2 μm, C18, 100 Å). A gradient elution of 5%–30% buffer B (0.1% formic acid in 80% acetonitrile; flow rate 0.6 μL/min) was used for 98 min. The MS data were acquired in data-dependent acquisition mode. Survey MS scans were acquired in the Orbitrap using a mass-to-charge ratio range of 300–1,500 with a resolution set to 70,000. The most intense ions per survey scan (top speed mode) were selected for collision-induced dissociation fragmentation, and the resulting fragments were analysed in the Orbitrap with a resolution of 30,000. Dynamic exclusion was employed with a 20 s window to prevent repetitive selection of the same peptide. The normalized collision energy for the HCD-MS2 experiments was set to 32%.

### LC–MS/MS data analysis

Raw MS data files were processed using Proteome Discoverer 2.1-Sequest software (Thermo Scientific). Features with only one charge or more than five charges were excluded from analysis. Only peptides with a Mascot score >30 and a *p* < 0.01 for the identified proteins were included for further quantitation. Proteins identified using at least one peptide were retained. MS/MS spectra were exported and processed using Mascot software (version 2.5.1, Matrix Science, London, United Kingdom) against the NCBI database (human reference sequence 2017-11-01) with the following search parameters: 10 ppm precursor mass tolerance, 0.05 Da fragment mass tolerance, and up to two missed cleavage sites allowed in trypsin digestion. Only highly confident peptide identifications (FDR ≤0.01) were imported into Progenesis software for further analysis. DEPs were identified using statistical criteria of a *t*-test *p*-value <0.05, a minimum of two peptides matched to a protein, and a fold change >1.5. Principal component analysis (PCA) was performed on the DEPs without missing values. A heatmap of the DEPs was generated using OmicShare software.

### Enzyme-linked immunosorbent assay

All urine samples were analysed in a blinded fashion with standards and the samples analysed in triplicate. The concentrations of inter-α-trypsin inhibitor heavy chain H1 fragment (ITIH1), recombinant programmed cell death protein 1 ligand 2 (PDCD1LG2), human NME1-NME2 complex (NME1-NME2), and chondroitin sulphate proteoglycan 4 (CSPG4) were quantified using a Human ITIH1 ELISA Kit (No. MM61243H1), Human PDCD1LG2 ELISA Kit (No. MM61263H1), Human NME1-NME2 ELISA Kit (No. MM61229H1), and Human CSPG4 ELISA Kit (No. MM6125H1) respectively. A standard curve was generated and used to determine the concentrations of ITIH1, PDCD1LG2, NME1-NME2, and CSPG4 in the samples.

### Statistical analyses

The IBM Statistical Package for the SPSS software was used for statistical analysis. Data were tested for normality and parametric tests were used for normally distributed data and nonparametric tests for data not normally distributed. Independent sample t-tests or Mann–Whitney U tests were used to determine the differences between groups with the appropriate test being chosen based on the normality of the data. All data are presented as the mean ± standard deviation (SD). *p* < 0.05 was considered statistically significant. For DEP analysis, we used an adjusted *p*-value based on multitest. Comparisons between LL, LH, HL, and HH groups were performed using non-parametric t-tests for routine blood test analyses and ELISA. Comparisons between the LC and HC groups were performed using parametric t-tests.

## Results

### Baseline characteristics of participants

**FIGURE 1:**
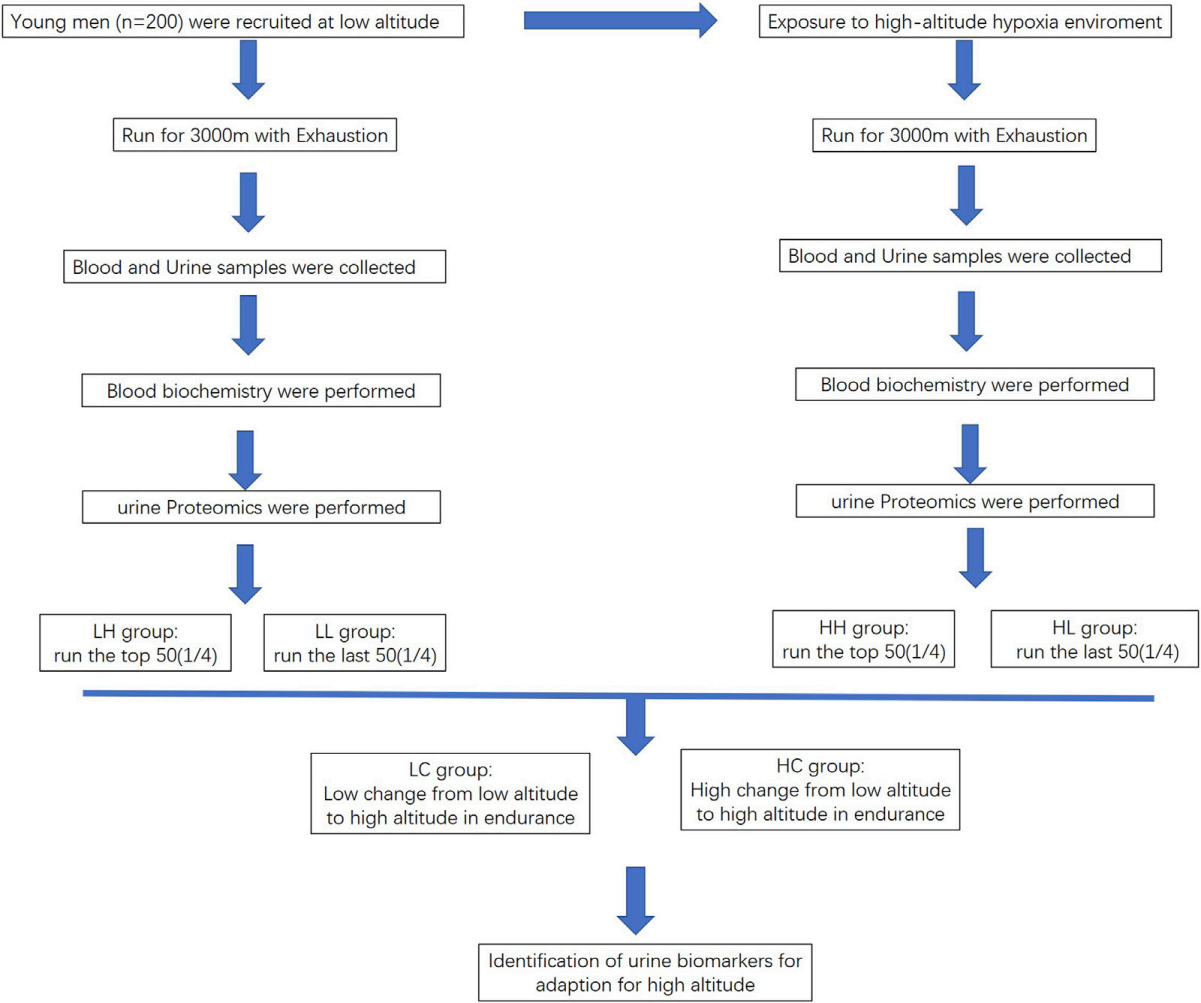
Flowchart outlining the process for identifying biomarkers for high endurance in high-altitude adaptation.

The baseline characteristics of all the participants are listed in Table 1. No significant differences were observed in sex, age, or height between the individuals. The weight of the HH and HL groups did not show significant differences, but some differences were noted at low altitude. The scores on the 3,000 m run were significantly higher for the individuals in the LH group than for those in the LL group (12′11” ± 22″ vs. 13′04” ± 18″, *p* < 0.0001) and for those in the HH group than those in the HL group (14′22” ± 39″ vs. 14′58” ± 46″, *p* < 0.0001) (Table 1; Figure 1).

**TABLE 1:** Baseline characteristics of the enrolled subjects.

	LH	LL	HH	HL	*p*-Value
**Sex**					
Male	50 (100%)	50 (100%)	50 (100%)	50 (100%)	NA
Female	0 (0%)	0 (0%)	0 (0%)	0 (0%)	
**Age(years)**					
Mean (SD)	20 (1.41)	20 (1.66)	20 (1.65)	20 (1.58)	P_12_ = 0.99; P_34_ = 0.99; P_13_ = 0.91; P_24_ = 0.95
Median [min,max]	20 [18,23]	20 [18,25]	20 [18,25]	20 [18,25]	
**Height(cm)**					
Mean (SD)	175.17 (6.58)	174.31 (5.58)	174.18 (6.21)	173.25 (5.21)	P_12_ = 0.89; P_34_ = 0.86; P_13_ = 0.84; P_24_ = 0.82
Median [min,max]	175 [162,190]	173.25 [161,188]	174.5 [161,188]	172 [160,188]	
**Weight(kg)**					
Mean (SD)	67.75 (7.23)	73.17 (10.06)	62.8 (6.94)	67.20 (8.58)	P_12_ = 0.009; P_34_ = 0.06; P_13_ = 0.02; P_24_ = 0.003
Median [min,max]	67.3 [52,82.3]	71.2 [58.1,104.5]	62.1 [51.4,77.4]	65.25 [54.3,89.9]	
**3000m(s)**					
Mean (SD)	12′11” (22″)	14′22” (39″)	13′04” (18″)	14′58” (46″)	P_12_ < 0.0001; P_34_ < 0.0001; P_13_ < 0.0001; P_24_ < 0.0001
Median [min,max]	12′16” [11′09″,12′39”]	14′14’ [13′40″,16′40”]	13′02” [12′20″,13′30”]	14′40” [14′20″,18′29”]	

### Routine blood tests of HH and HL groups

**FIGURE 2:**
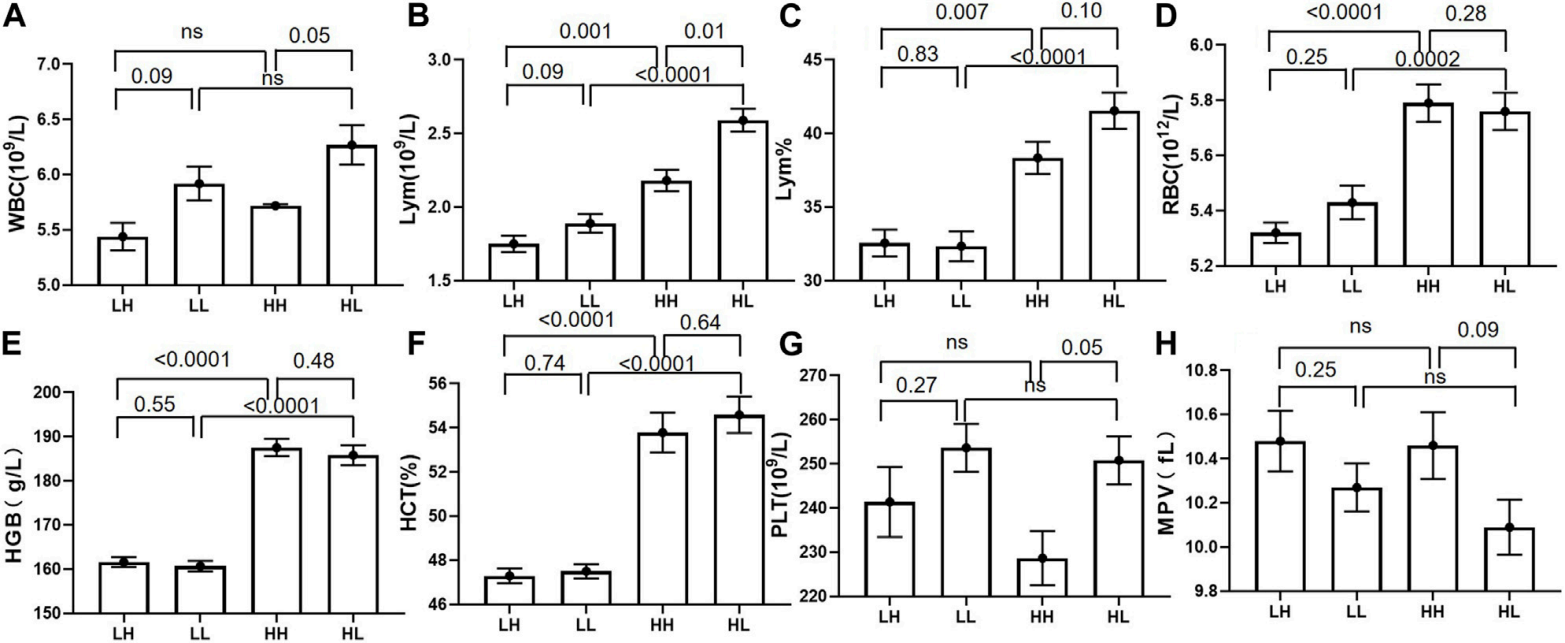
The routine blood test results for low-altitude with high stamina (LH) and low-altitude with low stamina (LL) groups at low altitude and high-altitude with high stamina (HH) and high-altitude with high stamina (HL) groups at high altitude. **(A, B, G, H)** White blood cell (WBC), lymphocyte (LYM), and platelet (PLT) counts in the HH group were significantly higher than those in the HL group. Mean platelet volume (MPV) was higher in the HH group than that in the HL group, however the difference was not significant. **(B–F)** The corresponding indexes including lymphocyte percentage (LYM%), haematocrit (HCT)%, and LYM, red blood cell (RBC), and haemoglobin (HGB) counts in HH and HL groups were significantly higher than those in the LH and LL groups.

The mean values for RBC, HGB, HCT, LYM, and LYM% were significantly higher at high altitude in the HH group (mean value 5.79 ± 0.48; 187.53 ± 13.94; 53.79 ± 6.39; 2.18 ± 0.52; and 38.34 ± 7.68) than that at low altitude in the LH group (mean value 5.32 ± 0.26; 161.6 ± 7.94; 47.3 ± 2.36; 1.75 ± 0.39; 32.56 ± 6.40) (*p* < 0.005). Similarly, the mean values for RBC, HGB, HCT, LYM, and LYM% were significantly higher at high altitude in the HL group (mean value 5.76 ± 0.48; 185.8 ± 16.11; 54.59 ± 5.83; 2.59 ± 0.54; 41.55 ± 8.68) than at low altitude in the LL group (mean value 5.43 ± 0.43; 160.7 ± 8.57; 47.5 ± 2.29; 1.89 ± 0.45; 32.34 ± 7.10) (*p* < 0.005). Furthermore, the concentrations of WBC, LYM, and PLT were significantly lower in the HH group (mean value 5.72 ± 0.99; 2.18 ± 0.52; 228.65 ± 43.26) than in the HL group (mean value 6.27 ± 1.25; 2.59 ± 0.54; 250.8 ± 38.42) (*p* < 0.05). Although the WBC, LYM, and PLT concentrations were lower in the LH group than in the LL group, the difference was not significant (Table 2; Figure 2).

**TABLE 2:** Results of routine blood tests in LH/LL groups at low altitude and HH/HL groups at high altitude.

	LH	LL	HH	HL	*p*-Value
WBC(10^9^/L)	5.43 ± 0.88	5.92 ± 1.09	5.72 ± 0.99	6.27 ± 1.25	P_12_ = 0.09; P_34_ = 0.05; P_13_ = 0.69; P_24_ = 0.57
Lym (10^9^/L)	1.75 ± 0.39	1.89 ± 0.45	2.18 ± 0.52	2.59 ± 0.54	P_12_ = 0.09; P_34_ = 0.01 P_13_ = 0.001; P_24_ < 0.0001
Lym%	32.56 ± 6.40	32.34 ± 7.10	38.34 ± 7.68	41.55 ± 8.68	P_12_ = 0.83; P_34_ = 0.10 P_13_ = 0.007; P_24_ < 0.0001
RBC(10^12^/L)	5.32 ± 0.26	5.43 ± 0.43	5.79 ± 0.48	5.76 ± 0.48	P_12_ = 0.25; P_34_ = 0.28 P_13_ < 0.0001; P_24_ = 0.0002
HGB (g/L)	161.6 ± 7.94	160.7 ± 8.57	187.53 ± 13.94	185.8 ± 16.11	P_12_ = 0.55; P_34_ = 0.48 P_13_ < 0.0001; P_24_ < 0.0001
HCT (%)	47.3 ± 2.36	47.5 ± 2.29	53.79 ± 6.39	54.59 ± 5.83	P_12_ = 0.74; P_34_ = 0.64 P_13_ < 0.0001; P_24_ < 0.0001
PLT (10^9^/L)	241.38 ± 56.19	253.63 ± 38.32	228.65 ± 43.26	250.8 ± 38.42	P_12_ = 0.27, P_34_ = 0.05 P_13_ = 0.69; P_24_ = 0.99
MPV(fL)	10.48 ± 0.97	10.27 ± 0.77	10.46 ± 1.07	10.09 ± 0.88	P_12_ = 0.25; P_34_ = 0.09 P_13_ = 0.99; P_24_ = 0.86

### Urine peptidome profiles of HH and Hl groups

**FIGURE 3:**
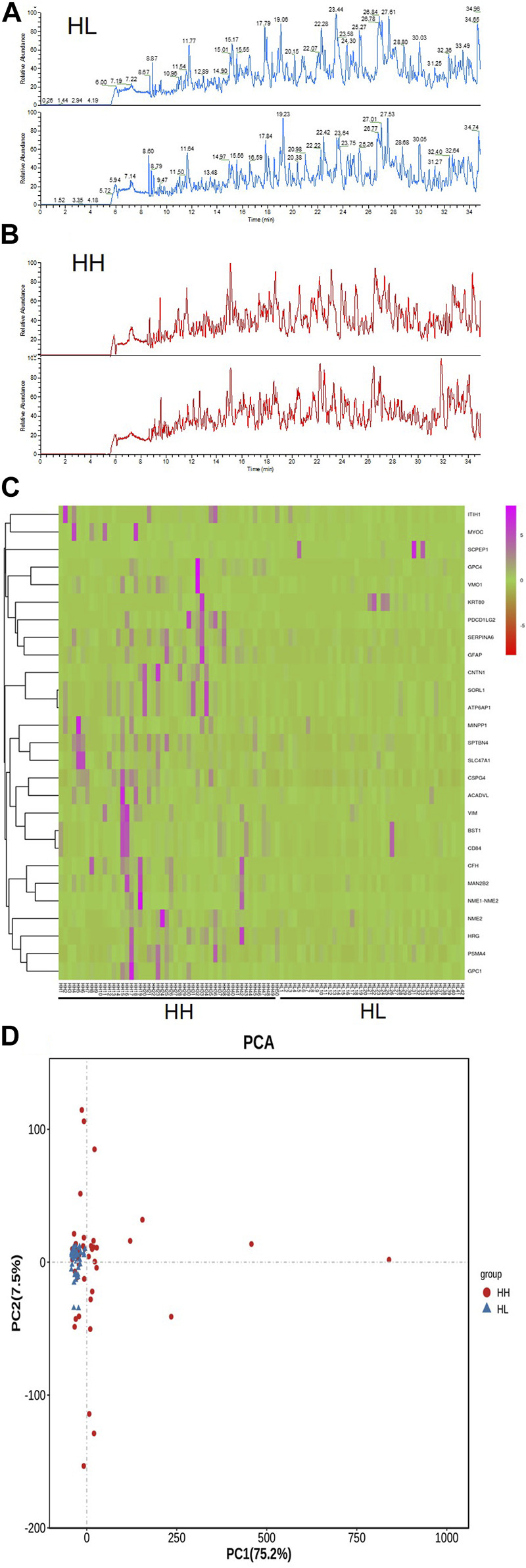
Reproducibility of mass spectra generated in individuals from the HH and HL groups, and comparative analysis of urine peptidome profiling between the HH and HL group samples collected at high altitude. **(A)** Representative mass spectra of an individual from the HL group (blue) and **(B)** representative mass spectra of an individual from the HH group (red), indicating low variability between replicates of this sample. **(C)** A heat-map representing the expression levels of proteins in the HH and HL groups. **(D)** Principal component analysis (PCA) of gene expression variations in HH and HL groups. A bivariate plot comparing the peptidome profiles of individuals from the HH (red) and HL (blue) groups.

The reproducibility and stability of the mass spectra results were evaluated using an HPLC-Orbitrap MS-based proteome platform, and the samples in the same group showed closely reproducible peaks (Figures 3A,B). The Heatmap of the HH and HL groups showed that most of the higher-expression proteins were concentrated in the HH group (Figure 3C). PCA demonstrated that the sample distribution pattern of the HH group was distinct from that of the HL group (Figure 3D).

### Identification of HH urea biomarkers

**FIGURE 4:**
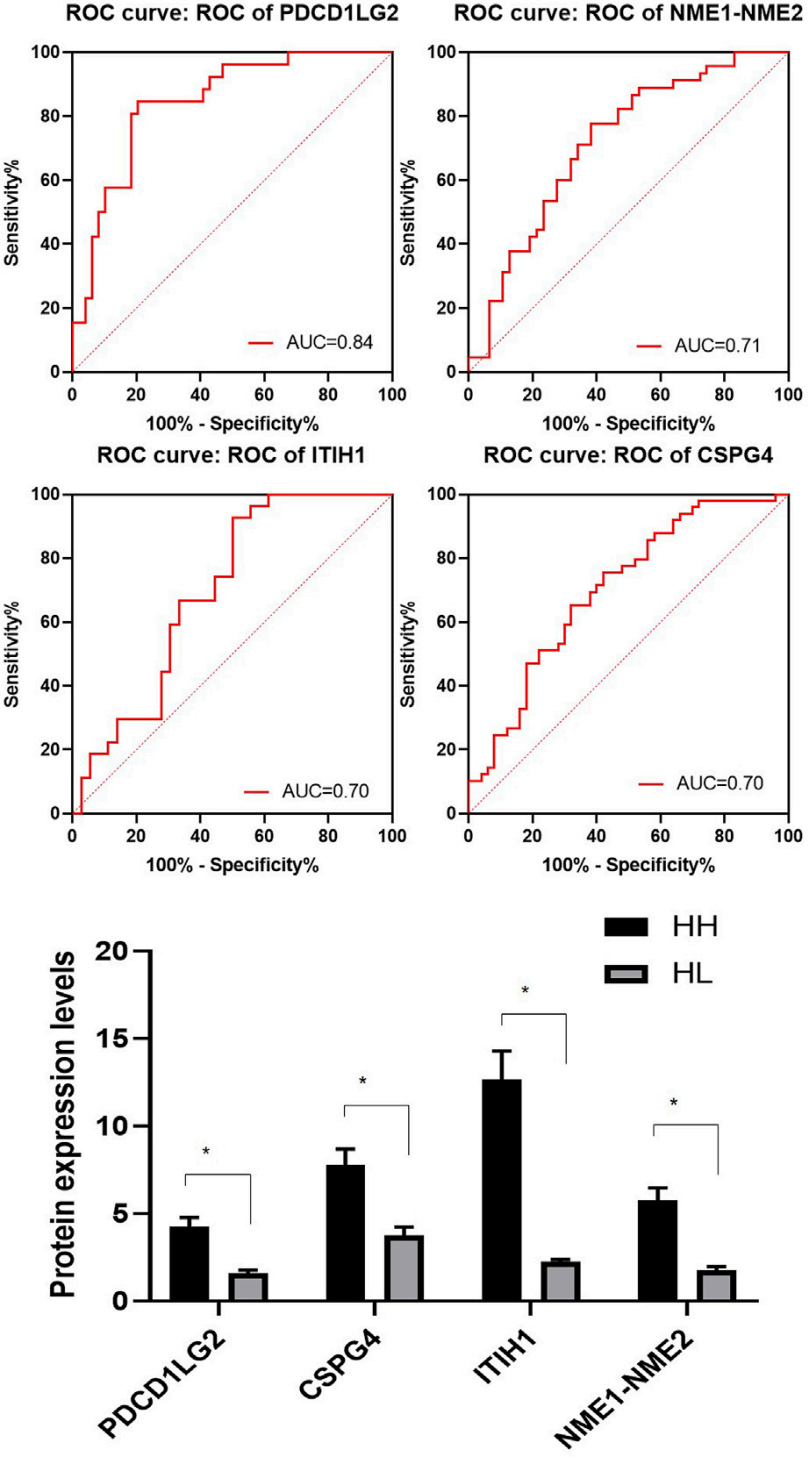
Representative spectra of four potential biomarkers in the HH group at high altitude. Receiver operating characteristic (ROC) curves for the four selected proteins with their area under the curve (AUC) values. The average expression levels of the four selected proteins in the HH (black) and HL samples (grey) and their *p* values expressed as mean ± SD.

A total of 144 different proteins were identified between the HH and HL groups with 33 of them showing fold changes >2 (*p* < 0.05). The four most significantly differential proteins are listed in Table 3 (*p* < 0.005, fold change >2), and their receiver operating characteristic (ROC) curves are shown in Figure 4. The area under the curve (AUC) values for these four proteins were 0.84, 0.71, 0.70, and 0.70 (Figure 4). All four proteins were upregulated in the HH group (Table 3; Figure 4).

**TABLE 3:** Mean levels of four differentially expressed proteins in HH and HL samples collected at high altitude.

Protein	HH	HL	Fold	*p*-value
PDCD1LG2	4.24 ± 3.78	1.60 ± 1.25	2.65	0.005
ITIH1	12.66 ± 11.50	2.25 ± 0.86	5.62	0.004
NME1-NME2	5.73 ± 5.08	1.75 ± 1.55	3.27	0.0005
CSPG4	6.65 ± 5.68	3.20 ± 3.16	2.07	0.0003

### Validation of protein expression in HH and HL groups

**FIGURE 5:**
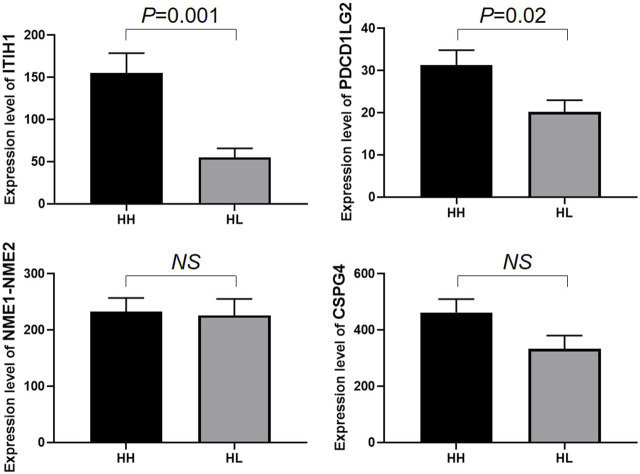
Enzyme-linked immunosorbent assay (ELISA) analysis of ITIH1, PDCD1LG2, NME1-NME2, and CSPG4 expression in the HH and HL groups. The y-axis represents protein expression levels (ng/mL) in different groups. Statistical analysis was performed using Prism 8.0 software. All data with *p* values are represented as the mean ± SD.

To validate the expression levels of PDCD1LG2, ITIH1, NME1-NME2, and CSPG4 obtained from the LC–MS/MS analysis, the urine concentrations of these proteins were further examined in 96 samples from HH and HL groups using ELISA. Urinary concentrations of the four proteins in the two groups are shown in Table 4 and Figure 5. The mean concentration of ITIH1 was 144.21 ± 12.64 in the HH group and 55.10 ± 40.11 ng/mL in the HL group (*p* = 0.001), indicating that ITIH1 was expressed at significantly higher levels in the HH group. Similarly, the mean concentration of PDCD1LG2 was 32.35 ± 22.40 ng/mL in the HH group and 20.14 ± 14.39 ng/mL in the HL group (*p* = 0.02), indicating that PDCD1LG2 was also expressed at significantly higher levels in the HH group. The urine concentrations of NME1-NME2 and CSPG4 were also higher in the HH group with mean concentrations of 232.99 ± 134.63 ng/mL and 443.06 ± 251.56 ng/mL, respectively, compared with that of the HL group with mean concentrations of 203.73 ± 123.12 ng/mL and 356.98 ± 278.80 ng/mL, respectively. This indicated that NME1-NME2 and CSPG4 were also expressed at higher levels in the HH group but the difference was not statistically significant (*p* > 0.05) (Table 4; Figure 5). These results were consistent with the findings of the UPLC-MS/MS analysis.

**TABLE 4:** Mean levels of four differentially expressed proteins verified by ELISA in HH and HL samples.

	HH	HL	*p*-value
ITIH1 (range) ng/mL	16.71-469.58	3.34-231.82	0.001
Mean ± Std	144.21 ± 126.64	55.10 ± 40.11	
PDCD1LG2 (range) ng/mL	2.08-83.13	2.08-53.66	0.02
Mean ± Std	32.35 ± 22.40	20.14 ± 14.39	
NME1-NME2 (range) ng/mL	46.87-524.21	32.18-465.46	>0.05
Mean ± Std	232.99 ± 134.63	203.73 ± 123.12	
CSPG4 (range) ng/mL	80.59-942.07	43.13-848.43	>0.05
Mean ± Std	443.06 ± 251.56	356.98 ± 278.80	

### Urine biomarkers filtrates of LC and HC groups

**FIGURE 6:**
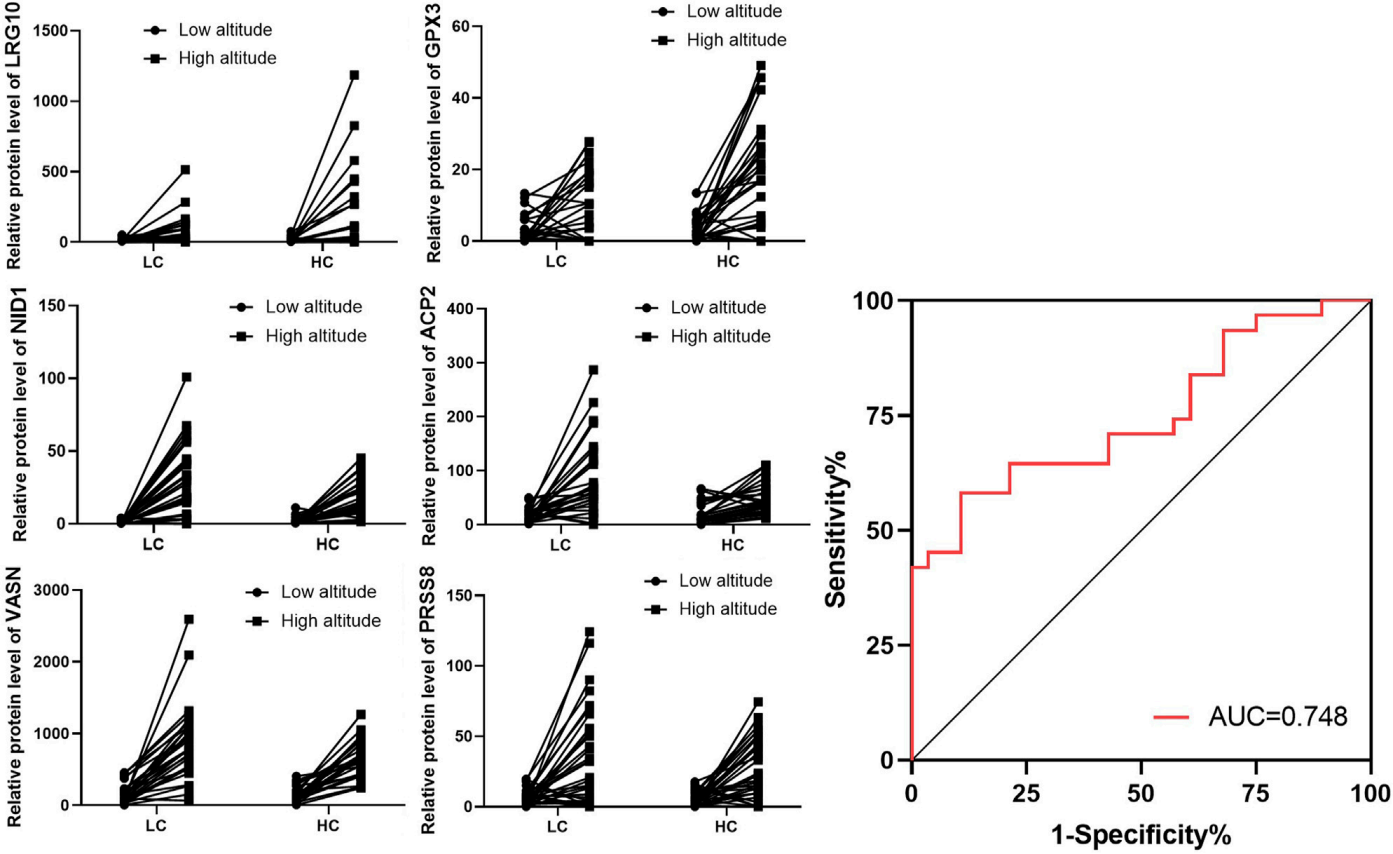
Expression of individual screened proteins for LC and HC groups at low and high altitudes. Expression of the six selected proteins in the LC and HC groups from low to high altitude. Combined Receiver operating characteristic (ROC) curve for all six selected proteins with their area under the curve (AUC) values.

The urine of individuals in the LC and HC groups were screened for biomarkers associated with changes in endurance. Results showed that six proteins were significantly upregulated in high altitude compared to low altitude. The six proteins were leucine rich alpha-2-glycoprotein 1 (LRG1), glutathione peroxidase 3 (GPX3), and decreases in protein expression of nidogen 1 (NID1), vasorin (VASN), acid phosphatase 2 (ACP2), and serine protease 8 (PRSS8). The ROC curve and AUC values were used to assess the combined diagnostic value of the six proteins for high stamina in high-altitude adaptation. The ROC curve is shown in Figure 6, and the combined AUC value for the six proteins was 0.748 (Figure 6).

### Validation of inflammation factor in LC and HC groups

**FIGURE 7:**
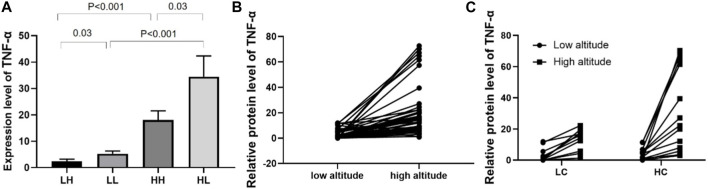
ELISA analysis of TNFα in different groups. **(A)** The expression of TNFα in HH/HL was significantly higher than in LH/LL group. **(B)** The expression of TNFα was significantly increased from low altitude to high altitude. **(C)** Individual expression of TNFα in the LC and HC groups from low to high altitude.

As the level of inflammation is an important factor in high-altitude adaptation, we validated the expression levels of tumour necrosis factor a (TNFα) in different groups using ELISA. The mean concentration of TNFα was 2.40 ± 0.76 in the LH group, 5.30 ± 1.06 ng/mL in the LL group (*p* = 0.03), 18.09 ± 3.47 in the HH group, and 34.47 ± 7.89 ng/mL in the HL group, indicating that TNFα was expressed at significantly higher levels in the HL group compared to the other groups. Increased expression of TNFα was also observed in low altitude to high altitude adaptation and LC to HC groups (Figure 7).

## Discussion

The study of high-altitude adaptation is a well-established area in the field of biological anthropology with a focus on the physiological effects of hypobaric hypoxia on high-altitude populations (Hartman-Ksycińska et al., 2016). However, few studies have explored the physical fitness of athletes during high-altitude adaptation. To the best of our knowledge, this study is the first to analyse urine proteomics in the HL/HH and LC/HC groups to investigate the adaptability of lowland athletes to high-altitude hypoxia with high performance. We have demonstrated the feasibility and safety of using proteomics to identify urine biomarkers. Four potential urinary protein biomarkers were identified in the HL and HH groups, and six combined urine protein biomarkers were screened from the LC and HC groups, which may play a role in maintaining high stamina during high-altitude adaption.

Routine blood tests revealed that the concentrations of HGB, RBC, and HCT levels in the high-altitude samples were significantly higher than those in low-altitude samples regardless of stamina levels. This is consistent with the response of lowland mammals to chronic hypoxia, which typically involves increased erythropoietic activity, leading to an increase in HGB, RBC, and HCT concentrations (Wagner et al., 2015). The moderate increase in HGB concentration observed in the high-stamina group may have contributed to the increased blood oxygen-carrying capacity and improved tissue oxygenation, helping these individuals to maintain high stamina during high-altitude adaptation (Yang et al., 2020). Additionally, the concentrations of WBC, LYM, PLT, and LYM% were lower in high-stamina samples than in low-stamina samples, with a greater difference observed between HH and HL groups than between LH and LL groups. These results show that the difference in protein expression between different stamina groups is more significant at high-altitudes than at low-altitudes. The lower expression of WBC, LYM, and LYM% in the HL group compared with that of the HH group suggests that soldiers with higher altitude running speeds have lower levels of inflammatory stress and better antioxidant capacity (Xiong et al., 2020). Kostrzewa et al. confirmed that the increase in WBC count after exercise is related to the immune response and is not solely caused by dehydration (Nowak et al., 2020). WBC counts often decline after prolonged training, and high altitudes are associated with an inflammatory response (Galun et al., 1987). In comparison with individuals with poor stamina, those with high stamina maintain a low inflammatory level to adapt to high altitudes. Additionally, we found that PLT was highly expressed in the HL samples. PLT plays a role in blood coagulation (Nader et al., 2021). In the past decade, many epidemiological studies have shown that high PLT expression is a major risk factor for cardiovascular disease (Picker et al., 2013). Additionally, blood coagulation glycoproteins are highly expressed in patients with high-altitude hypoxia (Yang et al., 2013). The present study suggests that the high expression levels of PLT in HL samples may lead to enhanced blood coagulation, resulting in low stamina and difficulty in adapting to high-altitude hypoxic environments.

In our urine peptidome study, we identified four DEPs that varied in levels between the HH and HL groups. All four proteins were upregulated in the HH group compared to the HL group, and their mean recognition capacity had an ROC value of ≥70%. The four potential urine biomarkers identified for HH were ITIH1, PDCD1LG2, NME1-NME2 and CSPG4. The expression levels of these four proteins were verified using ELISA in the HH and HL groups. ELISA results confirmed the upregulation of ITIH1 and PDCD1LG2 in the HH group. ITIH1 is a heavy chain of a serine protease inhibitor that may act as a carrier of hyaluronan or as a binding protein between hyaluronan and other matrix proteins, playing a role in inflammation and carcinogenesis (Hamm et al., 2008). ITIH1 contains a putative binding site for hyaluronic acid, a ubiquitous component of the extracellular matrix (ECM) (Scott et al., 2009). Therefore, ITIH1 could be involved in ECM stabilization (Huang et al., 1993). Thus, they accumulate in the vascular endothelium and may play a role in the stabilization of endothelial cells and ECM damaged by high-altitude hypoxia. Low expression of ITIH1 in HL urine samples may be a vital clue for the inability to adapt to high-altitude hypoxic environments as the conditions of vascular endothelial cells have been affected. PDCD1LG2 is involved in costimulatory signalling that is essential for T-cell proliferation and IFNG production in a PDCD1-independent manner. Interaction with PDCD1 inhibits T-cell proliferation by blocking cell cycle progression and cytokine production (Huang et al., 2020). PDCD1LG2 is highly expressed in the heart, placenta, pancreas, lung, and liver and weakly expressed in the spleen, lymph nodes, and thymus (Takamochi et al., 2022). It is upregulated by IFNG/IFN-gamma stimulation in monocytes and induced in dendritic cells grown from peripheral blood mononuclear cells with CSF2 and interleukin-4 (Yang et al., 2022). High expression of PDCD1LG2 in HH urine samples may signify its role in adaptation to high-altitude hypoxic environments with high endurance by inhibiting T-cell proliferation and maintaining low inflammation.

Furthermore, we identified six DEPs that varied between the LC and HC groups. All six proteins were upregulated in high altitude compared to low altitude with increase in the protein expression of LRG1 and GPX3 and decreases in the protein expression of NID1, VASN, ACP2, and PRSS8 in the LC group compared with that of the HC groups. LRG1 is a Rho-GTPase-activating protein that acts as a GTPase activator and has metal ion-binding activity (Lin M et al., 2022).Several studies have investigated the biological effects of LRG1 as a factor in inflammation, vascular growth regulation, cell adhesion, and cell viability (Liu TT et al., 2021). Therefore, lower LRG1 expression in the LC group compared with that in the HC group suggests that individuals with little change in running speed with change in altitude have lower levels of inflammatory stress and better antioxidant capacity. GPX3 is a component of the glutathione peroxidase-like protective system against oxidative damage (Reddy AT et al., 2018). Lower GPX3 expression in the LC group than in the HC group suggests that individuals with little change in altitude running speed have lower levels of ROS stress and better antioxidant capacity. NID1 is a sulphated glycoprotein that is widely distributed in basement membranes and tightly associated with laminin, which plays a role in cell–ECM interactions (Ferraro DA et al., 2019). The relatively low expression of NID1 in HC urine samples may indicate the inability to adapt to high-altitude hypoxic environments as the cell–ECM interaction conditions have been affected. VASN acts as an inhibitor of TGF-β signalling, which has been shown to exert anti-apoptotic effects, and hypoxia has been reported to stimulate a robust elevation in VASN expression (Choi JA et al., 2022). We observed an increase in VASN expression at high altitudes. The accumulation of VASN in the LC group is indicative of its role in adaptation to high altitudes through anti-apoptotic and anti-TGF-β activities. ACP2 belongs to the histidine acid phosphatase family and is a carrier of the growing fatty acid chain in fatty acid biosynthesis (Yi Z et al., 2019). The relatively low expression of ACP2 in HC urine samples may indicate its significant role in the individual’s inability to adapt to high-altitude hypoxic environments owing to alteration of fatty acid metabolism. PRSS8 is a potential regulator of epithelial sodium channel (ENaC) function, causing increased blood pressure (Shipway A et al., 2004). Reduced prostasin expression in the IBD mucosa is linked to the deterioration of local anti-inflammatory activity (Sugitani Y et al., 2020). The lower expression of PRSS8 in the HC group might be related to the deterioration of the local anti-inflammatory activity.

In conclusion, this study aimed to identify urine protein biomarkers for people with high stamina levels and good adaptability to high altitudes. Our findings indicate that WBC, lym, PLT, and lym% were significantly higher in HH individuals than in HL individuals. Additionally, urine proteomics analysis revealed several differentially expressed proteins between HH and HL samples, with ITIH1 and PDCD1LG2 being identified as potential biomarkers for HH. Furthermore, our analysis identified six proteins (LRG1, NID1, VASN, GPX3, ACP2, and PRSS8) as urine proteomic biomarkers for high stamina in high-altitude adaptation, and a combination of these proteins could potentially be used in the screening of individuals with high-altitude adaptability. The use of proteomics in this study offers a new approach to identifying biomarkers for screening individuals who can adapt to high altitudes with high stamina levels. We hope to expand this study by verifying these findings using larger sample sizes.

## Figures and Tables

**Table:** 

**HAPH**	high-altitude pulmonary hypertension
**LC group**	low change in endurance from low altitude to high altitude
**HC group**	high change in endurance from low altitude to high altitude
**HH group**	high-altitude with high stamina
**HL group**	high-altitude with low stamina
**HGB**	haemoglobin
**RBC**	red blood cell count
**WBC**	white blood cell count
**Lym**	lymphocyte count
**Neu**	neutrophil number
**Mon**	monocyte number
**Eos**	eosinophil number
**Bas**	basophil number
**Lym%**	lymphocyte percentage
**Mon%**	monocyte percentage
**Eos%**	eosinophil percentage
**Bas%**	basophil percentage
**HCT**	haematocrit
**MCV**	mean corpuscular volume
**RDW-CV**	red blood cell distribution width coefficient of variation
**RDW-SD**	red blood cell distribution width standard deviation
**PLT**	platelet count
**MPV**	mean platelet volume
**PDW**	platelet distribution width
**PCT**	platelet volume
**DEPs**	Differentially expressed proteins
**PCA**	Principal component analysis
**ITIH1**	inter-α-trypsin inhibitor heavy chain H1 fragment
**PDCD1LG2**	Recombinant Programmed Cell Death Protein 1 ligand 2
**NME1-NME2**	Human NME1-NME2 complex
**CSPG4**	chondroitin sulphate proteoglycan 4
**LRG1**	leucine rich alpha-2-glycoprotein 1
**GPX3**	glutathione peroxidase 3
**NID1**	nidogen 1
**VASN**	Vasorin
**ACP2**	acid phosphatase 2
**PRSS8**	serine protease 8
**ROC**	receiver operating characteristic curve
**AUC**	area under the curve
**FOT**	fraction of total
**TNF-a**	tumour necrosis factor

## Data Availability

The datasets presented in this study can be found in online repositories. The names of the repository/repositories and accession number(s) can be found below: http://proteomecentral.proteomexchange.org; PXD039791.
